# Spectrum and prognostic significance of parasitic infection in ART naïve HIV patients

**DOI:** 10.1186/1471-2334-14-S3-P28

**Published:** 2014-05-27

**Authors:** G Padmjakshi, S Saini

**Affiliations:** 1 Department of Microbiology, Rural Medical College, Loni, Maharashtra, India

## Background

It is a well-known fact that HIV/AIDS patients are vulnerable to many diseases which result in serve morbidity and mortality. The purpose of the study is to assess the prevalence and pattern of intestinal parasites in ART naïve patients.

## Methods

Field sampling started from July 2012 to July 2013 which included a total of 163 HIV seropositive patients. After taking an informed consent and recorded CD4 date, stool samples were collected for screening of parasites. Two stool samples were collected in two vials, one with formalin and other with polyvinyl alcohol (PVA). Saline wet mount and Lugols Iodine mount was done by direct microscopy and a formalin ether concentration method to detect ova, trophozoites, cysts, and larvae of intestinal parasites.

## Results

A total of 57 (34.9%) patients were found positive from 163 newly registered HIV patients. The prevalence of parasitic infection was more in the age group of 31-40 years in males, where as in females 21-30 years. The most common isolate was Hook worm (29, 50.8%) followed by Isospora (13, 22%) *Cryptosporidium* (9, 15.7%), *E. histolytica* (5, 8.7%), and *Giardia intestinalis* (1, 1.75%). Most of the cases were asymptomatic and the CD4 count was ≤ 350 mm^3^. Coccidian parasites always presented diarrhea and had a CD4 count of <200 mm^3^. In this study the most presenting symptoms were constipation followed by weight loss and anemia.

## Conclusion

This high spectrum of infection before ART concludes that early screening should be made mandatory and lower CD4 counts mostly associated with presence of coccidian parasites with diarrhea as presenting symptom.

